# Occupational burnout among medical staff in a designated infectious disease hospital in Lanzhou: differences by length of service

**DOI:** 10.3389/fpubh.2026.1980986

**Published:** 2026-10-05

**Authors:** Beina Mu, Yang Zhang, Jin Gui, Lexing Feng, Sheng Li

**Affiliations:** 1 The No. 1 People's Hospital of Lanzhou, Department of Ophthalmology No. 1, Lanzhou, Gansu, China; 2 School of Public Health, Gansu University of Traditional Chinese Medicine, Lanzhou, Gansu, China; 3 The No. 2 People's Hospital of Lanzhou, Department of Public Health, Lanzhou, Gansu, China

**Keywords:** designated infectious disease hospital, length of service, medical staff, occupational burnout, occupational health

## Abstract

**Objective:**

To investigate the current status of occupational burnout among medical staff with different lengths of service in a designated infectious disease hospital in Lanzhou, and to analyze inter-group differences so as to provide a basis for stratified interventions.

**Methods:**

In May 2025, a questionnaire survey was conducted among 290 on-duty medical staff of the hospital, and 278 valid questionnaires were returned (valid response rate: 95.86%). Occupational burnout was assessed with the Chinese revised version of the Maslach Burnout Inventory–Human Services Survey (MBI-HSS), and burnout level was classified according to the criteria proposed by Li Yongxin et al. The Kruskal-Wallis H test was used to compare groups based on measurement data; the Fisher-Freeman-Halton test was used to compare groups based on burnout levels; and Spearman's rank correlation analysis was used to examine the relationship between burnout levels and years of service.

**Results:**

The overall detection rate of occupational burnout was 66.91% (186/278). Total burnout scores did not differ significantly across groups (H = 8.956, *P* = 0.111). Scores on the diminished personal accomplishment dimension differed significantly among groups (*H* = 13.409, *P* = 0.020), with the 21–25-year group scoring higher than the 0–5-year group (adjusted *P* = 0.045). There were statistically significant differences in burnout levels between groups (*P* = 0.015), but no significant correlation was found between years of service and burnout levels (*r* = −0.016, *P* = 0.786); the 16–20-year group had the highest overall detection rate (83.33%), and the 21–25-year group had the highest proportion of severe burnout (15.38%).

**Conclusion:**

Occupational burnout among medical staff at this hospital exhibits a distribution pattern characterized by “relatively high incidence in the middle to late stages, with varying manifestations across different dimensions.” It is recommended that a tiered intervention be implemented for the group with 16 to 20 years of service, placing equal emphasis on career development support and psychological support.

## 1 Introduction

Burnout is a syndrome resulting from prolonged work-related stress that has not been successfully managed. It is primarily characterized by emotional exhaustion, psychological detachment from work or a negative attitude toward work, and a reduced sense of professional efficacy (1). In the healthcare sector, burnout has become a critical issue affecting the stability and development of the system. It not only directly harms the physical and mental health of healthcare workers, leading to emotional exhaustion, depression, and sleep disorders, but also undermines team effectiveness, exacerbates talent loss, and even affects the quality of medical services and patient safety (2).

In recent years, Sun Yanyan et al. (2), based on a multicenter survey of 22 hospitals across five provinces (cities) nationwide, reported that the detection rate of occupational burnout among healthcare workers in China was 58.0%. A meta-analysis by Wei Qixiu et al. (3), which included 12 cross-sectional studies (total sample size of 6,017), showed a pooled detection rate of 68% (95% CI: 60%−76%, I^2^ = 97.9%). Although there was significant heterogeneity among the studies, the detection rate of occupational burnout among healthcare workers in China remains at a high level. A large-scale survey of U.S. healthcare workers conducted by Rotenstein et al. (4) during the COVID-19 pandemic revealed a burnout rate as high as 49.9%, indicating that occupational burnout among healthcare workers has become a global issue.

Healthcare workers at designated infectious disease hospitals face an even higher risk of occupational burnout due to the unique nature of their work environment. They are chronically exposed to a high risk of infection, leading to the continuous accumulation of psychological stress (5); at the same time, due to the unique nature of the pathogens and patients, it is necessary to maintain a high level of vigilance and continuously update professional knowledge, which takes a tremendous toll on both physical and mental health(6). Given the inherently dangerous nature of their work, their burnout may be even more severe. However, current domestic research on occupational burnout among healthcare workers has largely focused on general hospitals, with significantly insufficient attention paid to this specific group working in specialized infectious disease hospitals(7).

Furthermore, existing research suggests that years of service are a significant factor influencing occupational burnout among healthcare workers(2); however, for healthcare workers in designated infectious disease hospitals, the specific characteristics of burnout differences across different years of service, their evolution patterns, and influencing factors have not yet been systematically elucidated. This has resulted in a lack of evidence-based, stage-specific, and precise psychological support and intervention strategies for this group. Therefore, this study aims to analyze the distribution patterns of occupational burnout among healthcare workers with varying years of service at a designated infectious disease hospital in Lanzhou, identify high-risk stages, and develop stratified intervention strategies, thereby providing empirical evidence to maintain the stability and effectiveness of the infectious disease prevention and control workforce.

## 2 Materials and methods

### 2.1 Survey participants

In May 2025, a comprehensive survey was conducted among all active medical staff holding physician or nurse licenses at a designated infectious disease hospital in Lanzhou. A total of 290 questionnaires were distributed, and 290 were returned. After excluding 12 invalid questionnaires, 278 valid questionnaires were retained, resulting in a response rate of 95.86% (278/290). This study was reviewed and approved by the hospital's Medical Ethics Committee (Approval No.: 2024111901). All participants provided written informed consent prior to enrollment.

### 2.2 Inclusion and exclusion criteria

Inclusion Criteria: (1) Active medical staff currently employed at a designated infectious disease hospital with at least 3 months of service at the hospital; (2) Participants must fully understand the study‘s objectives and procedures and voluntarily sign an informed consent form.

Exclusion Criteria: (1) External experts and retired personnel rehired on a part-time basis who regularly participate in clinical activities; (2) Those who are not on duty during the survey period. Meeting any one of the above criteria constitutes grounds for exclusion.

### 2.3 Research methods

This study used a questionnaire survey method. The survey instruments included a self-designed demographic questionnaire and a burnout scale. The assessment of burnout utilized a Chinese version (8) of the MBI-HSS (9), adapted to the Chinese cultural context. The scale consists of 22 items across three dimensions: emotional exhaustion (9 items), depersonalization (5 items), and low personal accomplishment (8 items). Each item was rated on a 7-point scale ranging from 0 to 6. The total scores for the emotional exhaustion, depersonalization, and low personal accomplishment dimensions were 0–54, 0–30, and 0–48, respectively; higher scores indicate a more severe level of burnout in that dimension. The total burnout score was calculated as the sum of the scores across the three dimensions. Following the method described by Maslach et al. (9), the participants' scores on the three dimensions of occupational burnout were ranked using SPSS 27.0 statistical software, and the values at the upper third of the distribution for each dimension were calculated as cutoff value. The following cutoff values were identified for each dimension: emotional exhaustion ≥ 29 points, depersonalization ≥14 points, and low personal accomplishment ≥24 points. Following the method of Li Yongxin et al. (10), participants with scores below the cutoff value on all three dimensions were classified as having no burnout, while those with scores at or above the cutoff in 1, 2, or 3 dimensions were classified as having mild, moderate, or severe burnout, respectively. In this study, the Cronbach's α coefficient for the scale was 0.794; the Cronbach's α coefficients for the emotional exhaustion, depersonalization, and low personal accomplishment dimensions were 0.910, 0.549, and 0.819, respectively. Years of work experience were divided into six groups at 5-year intervals, consistent with the grouping method used in similar studies.

### 2.4 Quality control

A preliminary survey was conducted prior to the formal survey, and the results showed that medical staff took an average of approximately 11 minutes to complete the questionnaire. The formal survey was conducted in May 2025 via the Wenjuanxing platform, with each WeChat account allowed to complete the questionnaire only once. With the approval of the relevant hospital departments, the questionnaire was distributed. The introduction to the questionnaire provided a detailed explanation of the survey's purpose, content, and completion requirements, emphasizing that responses would be anonymous and that no personal privacy information would be disclosed. Participants voluntarily participated after providing informed consent. After the survey concluded, questionnaires with all answers identical, those with completion times less than half the average duration of the preliminary survey (< 5.5 min), or those containing serious logical errors were deemed invalid and excluded. A total of 12 questionnaires were excluded to enhance data reliability.

### 2.5 Statistical analysis

Data analysis was performed using SPSS 27.0 software. According to the Shapiro-Wilk test, neither the total occupational burnout score nor the subscale scores followed a normal distribution; therefore, they were described using M (P25, P75). Intergroup comparisons were performed using the Kruskal-Wallis H test (df = 5); when overall differences were statistically significant, pairwise comparisons were further conducted using the Dunn test, and the results were corrected using the Bonferroni method (reported as corrected *P*-values). Categorical data were described in terms of numbers and percentages. For comparisons of burnout level across groups, the Fisher-Freeman-Halton test was used because the theoretical frequency in some cells was < 5 (minimum theoretical frequency < 1). Spearman's rank correlation analysis was employed to explore the association between years of service and the degree of occupational burnout. To control for the influence of confounding factors, binary logistic regression analysis was performed with the presence or absence of occupational burnout (0 = no burnout, 1 = burnout) as the dependent variable, including gender and professional title as control variables. Results are expressed as odds ratios (OR) and their 95% confidence intervals (CI). A two-sided *P*-value < 0.05 was considered statistically significant.

## 3 Result

### 3.1 Total burnout scores among healthcare workers in a designated infectious disease hospital by years of service

The results showed that the total burnout score for the 0–5 years group was 60.00 (51.50–71.50) points, the 6–10 years group 65.00 (55.00–71.00), the 11–15 years group 61.00 (56.00–69.50), the 16–20 years group 62.00 (57.00–73.00), the 21–25 years group 67.00 (59.00–80.00), and the ≥26 years group 59.00 (53.00–66.00); among these, the 21–25 years group had the highest total burnout score, while the ≥26 years group had the lowest. The Kruskal-Wallis H test revealed no statistically significant differences in the distribution of total burnout scores across years of service groups (*H* = 8.956, df = 5, *P* = 0.111, ε^2^ = 0.032).

### 3.2 Scores on various dimensions of occupational burnout among healthcare workers in a designated infectious disease hospital, by years of service

The results showed that, on the emotional exhaustion dimension, healthcare workers with 16–20 years of service exhibited relatively higher levels of burnout compared to other groups. On the depersonalization dimension, healthcare workers with 6–10 years of service exhibited higher scores compared to other service year groups. Specifically, the median depersonalization score for the 6–10-year group was 14.00, which reached the cutoff value for this dimension (≥14). The differences in scores across these two dimensions were not statistically significant (H values of 10.717 and 9.047, respectively; *P* > 0.05). Regarding the low personal sense of accomplishment dimension, there were statistically significant differences among healthcare workers with different years of service (*H* = 13.409, df = 5, *P* = 0.020). Further *post-hoc* pairwise comparisons using the Dunn test, corrected with the Bonferroni method, revealed that the difference in low personal sense of accomplishment scores between the 0–5 and 21–25 years groups was statistically significant (Z = −2.971, corrected *P* = 0.045), while differences between all other groups were not significant (all *P*-values > 0.05). Compared with other service duration groups, healthcare workers with 11–15, 16–20, and 21–25 years of service exhibited relatively higher levels of burnout on this dimension, as shown in Table 1.

**Table 1:** Median (P_25_, P_75_) scores for each dimension of occupational burnout among healthcare workers in a designated infectious disease hospital by years of service.

Years of service	*n*	Emotional exhaustion	Depersonalization	Low personal accomplishment
0~5	55	25.00 (22.00~33.00)	11.00 (9.00~15.00)	19.00 (14.00~23.00)
6~10	82	27.00 (23.00~32.00)	14.00 (10.00~15.00)	21.00 (16.00~26.00)
11~15	32	24.00 (19.25~28.00)	11.00 (9.00~15.00)	24.00 (16.25~32.00)
16~20	30	29.00 (23.25~35.00)	11.50 (7.50~15.75)	24.00 (18.75~31.50)
21~25	26	27.00 (25.00~34.25)	13.50 (10.00~16.25)	24.00 (19.75~41.50)
≥26	53	25.00 (20.00~30.00)	11.00 (9.00~14.00)	22.00 (18.00~29.50)
H value		10.717	9.047	13.409
*P* value		0.057	0.107	0.020

### 3.3 Detection rate of burnout among healthcare workers in a designated infectious disease hospital by years of service

Among the 278 healthcare workers in this study, the overall detection rate of occupational burnout was 66.91% (186/278). Based on the cutoff values and the classification method proposed by Li Yongxin et al., the healthcare workers were divided into four categories: no burnout, mild burnout, moderate burnout, and severe burnout. Since the theoretical frequencies in each cell of the severe burnout column in the contingency table were all < 5 (with the minimum theoretical frequency < 1), the conditions for the chi-square test were not met. Therefore, the Fisher-Freeman-Halton test was used to compare differences between groups. The results showed that there were statistically significant differences in the distribution of occupational burnout level among healthcare workers in the designated infectious disease hospital with different years of service (*P* = 0.015). To control for the influence of confounding factors on the risk of occupational burnout, a binary logistic regression analysis was conducted with the presence or absence of occupational burnout (0 = no burnout, 1 = burnout) as the dependent variable, using the 0–5-year group as the reference group, and incorporating gender and professional title as control variables. The Hosmer-Lemeshow test indicated that the model fit well (χ^2^ = 3.999, df = 7, *P* = 0.780). The results showed that the risk of occupational burnout in the 16–20-year group was 3.007 times that of the 0–5-year group (OR = 3.007, 95% CI: 0.972–9.302, *P* = 0.056), approaching statistical significance; the risk of occupational burnout in the 21–25-year group was 1.483 times that of the 0–5-year group (OR = 1.483, 95% CI: 0.530–4.154, *P* = 0.453), with no statistically significant difference. Neither gender nor professional title showed a significant effect in this model (*P* > 0.05). Further analysis using Spearman's rank correlation revealed that the level of occupational burnout did not show a linear trend with increasing years of service (*r* = −0.016, *P* = 0.786). Although the linear trend was not significant, descriptive results suggested that the 16–20-year group and the 21–25-year group exhibited different patterns of burnout. Therefore, years of service were further grouped into three categories to observe distribution patterns: early ( ≤ 10 years), middle (11–25 years), and late (≥26 years). The burnout detection rate in the middle group (73.9%) was higher than that in the early group (65.0%) and the late group (60.4%), showing a trend of first increasing and then decreasing; however, the differences among the three groups were not statistically significant (χ^2^ = 3.178, *P* = 0.204). In terms of specific composition, the proportion of those without burnout was lowest in the 16–20-year group (16.67%) and highest in the 0–5-year group (43.64%). Mild burnout was the most common type across all groups, with the highest proportion observed in the 16–20-year group (56.67%). The proportion of severe burnout was highest in the 21–25-year group (15.38%), significantly higher than in all other groups. The overall occupational burnout detection rate was highest in the 16–20-year group (83.33%), indicating that burnout is prevalent among healthcare workers with this length of service, followed by the 6–10-year group (70.73%), as shown in Table 2.

**Table 2:** Proportions and detection rates of burnout level among healthcare workers by years of service.

Years of service	*n*	No burnout		Mild burnout		Moderate burnout		Severe burnout		occupational burnout	
		Detected cases	Detection rate	Detected cases	Detection rate	Detected cases	Detection rate	Detected cases	Detection rate	Detected cases	Detection rate
0~5	55	24	43.64%	15	27.27%	15	27.27%	1	1.82%	31	56.36%
6~10	82	24	29.27%	32	39.02%	25	30.49%	1	1.22%	58	70.73%
11~15	32	10	31.25%	17	53.13%	4	12.50%	1	3.13%	22	68.75%
16~20	30	5	16.67%	17	56.67%	6	20.00%	2	6.67%	25	83.33%
21~25	26	8	30.77%	9	34.62%	5	19.23%	4	15.38%	18	69.23%
≥26	53	21	39.62%	24	45.28%	7	13.21%	1	1.89%	32	60.38%
Total	278	92	33.09%	114	41.01%	62	22.30%	10	3.60%	186	66.91%

## 4 Discussion

### 4.1 Occupational burnout among healthcare workers in a designated infectious disease hospital shows a distribution trend of relatively higher detection rate in the middle and later career stages

This study found that there was no statistically significant difference in total burnout scores among healthcare workers with different years of service, nor did the level of burnout show a linear trend with years of service; however, there were significant differences in the distribution of burnout levels across groups. Further analysis of scores for each dimension revealed distinct distribution patterns across the different years-of-service groups: the emotional exhaustion dimension had the highest score in the 16–20-year group (median: 29.00), the depersonalization dimension had the highest score in the 6–10-year group (median: 14.00), and the low personal accomplishment dimension remained at a relatively high level across the three mid-to-late career groups (11–15, 16–20, and 21–25 years; all with a median of 24.00), with the 21–25-year group scoring significantly higher than the 0–5-year group. These distinct distribution patterns across the three dimensions indicate that the impact of years of service on occupational burnout is not a monotonically increasing or decreasing trend, but rather that each dimension follows its own trajectory. The manifestations of occupational burnout vary not only among different individuals but also across different stages of the same individual's career. Combined with the findings that the 16–20-year group had the highest overall detection rate (83.33%, with mild burnout predominating) and the 21–25-year group had the highest proportion of severe burnout (15.38%), these results suggest that the impact of years of service on occupational burnout does not simply reflect a net increase or decrease in overall burden, but rather exhibits a distribution trend with relatively higher detection rate in the middle and later stages of a career. After further adjusting for confounding factors such as gender and professional title, binary logistic regression analysis showed that the risk of burnout in the 16–20-year group remained 3.007 times that of the 0–5-year group (*P* = 0.056), indicating that this group still exhibits a higher tendency toward burnout risk even after excluding confounding factors. Although this result did not reach statistical significance in the traditional sense, when combined with the overall detection rate of 83.33% in this group and the effect size of OR = 3.007, the trend is noteworthy. Healthcare professionals with 16–20 years of service should be prioritized as a key target group for intervention.

### 4.2 Severe occupational burnout among healthcare workers in a designated infectious disease hospital

The findings of this study are generally consistent with the results of surveys conducted both domestically and internationally among healthcare workers in designated infectious disease hospitals. The burnout detection rate among HIV/AIDS healthcare workers in Zambia was 51% (11), while in public tuberculosis hospitals in South Korea, the rates for nurses and physicians were 67.3% and 57.7%, respectively (12). Domestic studies have also shown that the burnout detection rate among human immunodeficiency virus/acquired immunodeficiency syndrome (HIV/AIDS) healthcare workers was 76.9% (13), while the rates among tuberculosis prevention and control personnel in Beijing and Yunnan Province were 62.7% (14) and 70.63% (15), respectively. The data for healthcare workers in each years-of-service group in this study fall within this broad range, further confirming that healthcare workers in infectious disease settings are a high-risk group for occupational burnout. It should be noted that the assessment tools and burnout diagnostic criteria used in the aforementioned studies are not entirely consistent, so cross-study comparisons of detection rates can only serve as a rough reference. Furthermore, recent studies using Li Yongxin's criteria have shown that burnout levels vary across different healthcare worker groups. For example, among primary-care healthcare workers in Taicang City, the burnout detection rate for those with 10–20 years of service was 61.31% (16). In contrast, the burnout levels observed among healthcare workers at designated infectious disease hospitals in the middle and later stages of their careers were more pronounced in this study: the overall detection rate in the 16–20-year service group reached 83.33%, and the proportion of those experiencing severe burnout in the 21–25-year service group was as high as 15.38%. This suggests that, compared to primary-care healthcare workers, healthcare workers in infectious disease settings not only experience higher rates of burnout in the middle of their careers but also show a more pronounced tendency for burnout to progress to severe levels, reflecting the unique nature and severity of the occupational stress faced by this group.

### 4.3 Mechanisms of the mid-to-late career burnout peak among healthcare workers in a designated infectious disease hospital

Occupational burnout does not accumulate linearly with years of service; rather, the overall detection rate peaks among those with 16 to 20 years of service, showing a relatively high incidence in the mid-to-late career stages, with the mechanisms varying across different years-of-service groups. The group with 16 to 20 years of service had the highest overall detection rate (83.33%), primarily due to the combined pressure of career development bottlenecks and family responsibilities. A study by Zhou Yiqin et al. (17) points out that healthcare professionals in this stage consistently face difficulties regarding job promotions and repetitive work tasks, leading to a decline in professional novelty. At the same time, their medical responsibilities and managerial pressures are increasing, making it difficult to enhance their sense of professional accomplishment. Additionally, individuals in this age group are often at the peak of their family responsibilities—raising children and caring for older adult parents—and the combination of occupational stress and family burdens tends to create a compounding effect, exacerbating work-family conflict and thereby driving up the overall detection rate within this work experience range.

A further comparison of the composition of burnout between the two groups revealed that the 16–20-year group had a higher proportion of mild burnout (56.67%), while the proportion of severe burnout in the 21–25-year group jumped to 15.38%. This difference suggests that the shift from widespread mild burnout among those with 16–20 years of service to severe burnout among those with 21–25 years of service may reflect a cumulative process of transition from general mild feelings of burnout to severe psychological exhaustion. This transition is moderated by organizational support and individual coping strategies (18, 19).

The group with 21–25 years of service in this study showed a markedly high proportion of severe burnout (15.38%), which is closely associated with the accumulation of long-term stress and the compounding pressures of multiple roles. Although healthcare workers in this stage are technically proficient, as noted by Chai Ling et al. (20), they often shoulder heavier clinical responsibilities, as well as teaching and research duties. If there is a significant gap between career development expectations and reality, it can easily lead to a decline in self-efficacy and a gradual indifference toward work and patients, exacerbating depersonalization. Furthermore, insufficient organizational support and workplace interpersonal relationships are closely linked to levels of occupational burnout. A study by Li Xia et al. (21) showed that strong peer support can effectively alleviate occupational burnout, whereas its absence weakens this protective effect; Qu Ge et al. (22) pointed out that a negative work environment exacerbates emotional exhaustion among senior staff. Chai Ling et al. (20) further emphasized that insufficient organizational support and unclear career advancement pathways amplify the dual pressures of inadequate career development and social support, ultimately resulting in an exceptionally high detection rate of burnout among healthcare workers with 21 to 25 years of service.

Low personal accomplishment was the only dimension in this study that showed significant differences between groups, particularly in the group with 21–25 years of service, reflecting the unique nature of infectious disease prevention and control work. Studies by Li Ling (15) and Cui Jiali (23) indicate that characteristics such as prolonged exposure to critically ill patients, high occupational exposure risks, relatively low public attention, and delayed and difficult-to-quantify work outcomes can easily undermine the sense of professional value among infectious disease healthcare workers with long tenure. A large-scale survey of school public health staff in the United States during the COVID-19 pandemic conducted by Merkle et al. (24) also showed that insufficient workplace support was associated with high-pressure public health responsibilities and adverse psychological outcomes such as depression and anxiety, suggesting that the mental health of those who undertake high-risk prevention and control tasks over the long term without organizational support is more vulnerable. These characteristics align closely with the group in this study with 21–25 years of service—most of whom have long been engaged in infectious disease prevention and control—and collectively confirm that the nature of prevention and control positions is associated with low personal accomplishment.

It is worth noting that the total burnout score and overall detection rate in the group with ≥26 years of service were the lowest among all groups, showing a marked decline. This phenomenon may be driven by multiple mechanisms: First, survivor bias may be at play; individuals with higher levels of burnout may have already left the group by resigning, transferring to other positions, or withdrawing from frontline clinical work, leaving behind those who are generally well-adapted and have a high level of professional identity (25). Second, the professional roles of senior staff tend to stabilize, with some having assumed managerial or teaching positions (25, 26). They enjoy greater autonomy and control over resources, and as the burden of family responsibilities has largely eased, work-family conflict is alleviated (27). Third, coping strategies and emotional regulation skills developed over time may also buffer occupational stress (18). Since this study employs a cross-sectional design, it is unable to distinguish between the aforementioned age effect, cohort effect, and survivor effect; this explanation requires validation through longitudinal cohort studies.

Furthermore, the median depersonalization score for the 6–10-year group has reached the cutoff value, suggesting that depersonalization among healthcare workers in the early to mid-stages of their careers should not be overlooked. Healthcare workers in this stage often experience peak clinical workload and frequent night shifts, and the gap between their initial career aspirations and the realities of their work gradually becomes apparent. Previous studies have confirmed a significant positive correlation between work stress and depersonalization (28, 29). Healthcare workers with lower professional titles, due to limited career advancement pathways and a significant gap between their career expectations and actual rewards, have depersonalization scores significantly higher than those with higher professional titles (30). Factors such as high frequency of night shifts and heavy clinical workloads further exacerbate the continuous depletion of emotional resources. When organizational support is insufficient to replenish depleted psychological resources, emotional detachment becomes a defensive strategy for individuals to cope with sustained stress. Long-term, high-intensity interpersonal care demands can easily lead them to adopt emotional detachment as a self-protection strategy. A prevention-oriented approach is recommended for this group; through measures such as reasonable scheduling, job rotation, mentorship support, and training in compassionate care, mild burnout can be prevented from progressing to moderate or severe levels.

In summary, based on an analysis of differences across years of service, this study reveals a trend of relatively high incidence of mid-to-late-stage occupational burnout among healthcare workers in the designated infectious disease hospital. To effectively address this situation, it is recommended that hospitals implement tiered, targeted intervention measures. Interventions should prioritize the group with 16 to 20 years of service, where occupational burnout is most prevalent, by establishing a dual-track system that simultaneously enhances both career development and psychological support. Specifically, for the 16–20-year group, where mild burnout predominates, the focus should be on career development incentives: establishing “dual-track” promotion pathways for clinical and managerial roles, providing opportunities for continuing education, research funding, and mentoring platforms, and supplementing these with job rotation and flexible scheduling to revitalize their interest in work and sense of accomplishment. For the 21–25-year group, which has the highest proportion of severe burnout, the focus should be on individual psychological intervention and organizational support: leveraging the Employee Assistance Program (EAP) to routinely offer psychological services such as mindfulness-based stress reduction training and Balint groups, while providing one-on-one counseling and referrals for those experiencing moderate to severe burnout. For the 6–10-year group, early intervention should focus on mentorship and career planning. This group is at a stage where the gap between career expectations and reality becomes apparent, and tendencies toward emotional detachment are beginning to emerge. By assigning experienced career mentors to early- and mid-career staff and providing regular career development counseling, reasonable career expectations can be established and emotional detachment can be prevented from progressing to moderate or severe levels. Second, fostering a positive organizational and social sense of recognition is equally important. Hospital administrators should strive to create a supportive work environment, reasonably allocate work hours and responsibilities, and enhance healthcare workers' professional pride and sense of social recognition by actively promoting the value of infectious disease prevention and control efforts. Finally, healthcare workers are encouraged to maintain a regular exercise routine and get adequate sleep, treating daily health management as a fundamental measure to prevent occupational burnout.

#### 4.3.1 Limitations of this study

(1) This study is a cross-sectional survey that can only reflect burnout at a specific point in time; it does not allow for longitudinal observation of the dynamic changes in occupational burnout over the course of one's career, nor can it distinguish between age effects, cohort effects, and survivor effects. (2) The sample was drawn exclusively from a designated infectious disease hospital in Lanzhou, and the sample size was limited; therefore, caution is required when generalizing the findings. (3) The data were collected via self-report questionnaires and may have been influenced by subjective factors such as social desirability and recent emotional states. (4) Multivariate analysis showed that the risk of burnout in the 16–20-year group reached only marginal significance (*P* = 0.056), which may be related to the limited sample size; this finding requires further validation in a larger sample. Furthermore, the sample size in some groups was small (only 26 participants in the 21–25-year group, with only 4 cases of severe burnout), resulting in low statistical power for intergroup comparisons, which may have masked some true differences. (5)The regression model only adjusted for gender and professional title, and other potential confounders (e.g., department, educational level, and organizational support) were not included, which may have left residual confounding. (6) The Cronbach's α coefficient (0.549) for the depersonalization dimension of the MBI scale is lower than the generally recommended standard of 0.70. However, this phenomenon is not unique to this study. A meta-analysis of MBI reliability by Aguayo-Estremera et al. (31), which included 69 studies and 37,160 participants, found that the average Cronbach's α coefficient for the depersonalization dimension ranged from 0.685 to 0.706, with the lower limit of the 95% prediction interval as low as 0.485. This indicates that the reliability of this dimension varies significantly across different samples, and may even fall to unacceptable levels in some samples. Yslado Méndez et al. (32) reported similar results in a validation study involving 303 Peruvian healthcare workers, in which the Cronbach's α for the depersonalization dimension was only 0.574 and the omega coefficient was 0.492, and the four items in this dimension showed insufficient discriminant validity. This may be related to the small number of items in this dimension (only 5) and the inclusion of sensitive content, such as attitudes toward patients. (7) Since the questionnaire was distributed with the assistance of relevant hospital departments, there may be some social desirability bias.

## Data Availability

The raw data supporting the conclusions of this article will be made available by the authors, without undue reservation.
